# A cost function analysis of child health services in four districts in Malawi

**DOI:** 10.1186/1478-7547-11-10

**Published:** 2013-05-10

**Authors:** Benjamin Johns, Spy Munthali, Damian G Walker, Winford Masanjala, David Bishai

**Affiliations:** 1 Department of International Health, Johns Hopkins Bloomberg School of Public Health, Institute for International Programs, 615 N. Wolfe Street, Baltimore, MD 21205, USA; 2 Department of Economics, University of Malawi, Chancellor College, Zomba, Malawi; 3 Department of Population, Johns Hopkins Bloomberg School of Public Health, Family and Reproductive Health, 615 N. Wolfe Street, Baltimore, MD 21205, USA

**Keywords:** Cost function, Efficiency, Malawi, Primary health care

## Abstract

**Background:**

Recent analyses show that donor funding for child health is increasing, but little information is available on actual costs to deliver child health care services. Understanding how unit costs scale with service volume in Malawi can help planners allocate budgets as health services expand.

**Methods:**

Data on facility level inputs and outputs were collected at 24 health centres in four districts of Malawi visiting a random sample of government and a convenience sample of Christian Health Association of Malawi (CHAM) health centres. In the cost function, total outputs, quality, facility ownership, average salaries and case mix are used to predict total cost. Regression analysis identifies marginal cost as the coefficient relating cost to service volume intensity.

**Results:**

The marginal cost per patient seen for all health centres surveyed was US$ 0.82 per additional patient visit. Average cost was US$ 7.16 (95% CI: 5.24 to 9.08) at government facilities and US$ 10.36 (95% CI: 4.92 to 15.80) at CHAM facilities per child seen for any service. The first-line anti-malarial drug accounted for over 30% of costs, on average, at government health centres. Donors directly financed 40% and 21% of costs at government and CHAM health centres, respectively. The regression models indicate higher total costs are associated with a greater number of outpatient visits but that many health centres are not providing services at optimal volume given their inputs. They also indicate that CHAM facilities have higher costs than government facilities for similar levels of utilization.

**Conclusions:**

We conclude by discussing ways in which efficiency may be improved at health centres. The first option, increasing the total number of patients seen, appears difficult given existing high levels of child utilization; increasing the volume of adult patients may help spread fixed and semi-fixed costs. A second option, improving the quality of services, also presents difficulties but could also usefully improve performance.

## Background

Recent data indicate uneven progress in reaching the fourth Millennium Development Goal to reduce mortality in children under five (U5s) by two-thirds [1]. Although recent analyses have shown that donor funding for child health is increasing [2], little information is available on what countries themselves pay for child health [3], and thus data are lacking on the total amount of resources devoted to child health. Lacking these data, recent estimates of resource needs for child health rely on modelling current resources based on coverage rates and treatment protocols, or simply modelling the total costs needed if interventions were delivered at optimal quality [4-8]. Estimates of the amount of resources devoted to child health are important both for analyses of current resource allocations, such as national health accounts or sub-accounts, and for estimates of resources needed to achieve mortality reduction goals.

Recently, countries, including Malawi, have adopted and are scaling up community-based case management of childhood diseases [9], which is intended to address some of the shortcomings of previous implementation designs by moving care closer to households [10]. In Malawi, health surveillance assistants perform community-based case management outside of health facilities. However, data on the costs of this new pathway are limited [11], and its effects on the costs of current health services is lacking [12]. Data on costs of child health services and the determinants of those costs are an important component of assessing the cost-effectiveness of community-based case management [10,12,13]. This study does not assess questions related to community-based case management, but serves as a baseline for further data collection and analysis.

Analysis of the costs of health interventions ideally would include the marginal costs of expanding services, coupled with factors affecting costs, such as quality, case mix, and scale [14]. In this paper, we examine the costs of delivering child health services in 4 districts of Malawi at the start of the community-based case management programme for U5s. This analysis will help policymakers understand the cost structure of child health services and will provide a baseline for determining how costs change over time and as the new programme expands.

## Methods

The cost function specification and results refer to the costs and outputs specific to child health services. We use the terms total, average, marginal cost and other terms throughout this paper to refer to the total, average, or marginal costs for child health services.

### Setting

Malawi’s health budget operates using a sector-wide approach (SWAp), where government and donor funds are pooled and distributed to districts^9^. Districts have broad control over budgets in Malawi’s decentralized health system. In principle, the catchment population of health facilities determines budgets, but funding levels may differ by district. In practice, health facilities order drugs from the central medical stores and thus costs are responsive to utilization. In addition to government health facilities, the Christian Hospital Association of Malawi (CHAM) operates health centres, which charge clients for services, in coordination and cooperation with the government.

Some donors operate, purchase, or distribute commodities outside the SWAp; for example, The Global Fund to Fight AIDS, Tuberculosis, and Malaria (GFATM) provides funds to purchase anti-malarial drugs, The Global Alliance for Vaccines and Immunisation (GAVI) purchases pentavalent vaccines, and some donors may supply famine relief, outbreak control, and other measures.

### Data collection

We collected data in four districts, all from central or southern Malawi, purposively selected in consultation with Ministry of Health staff. Retrospective data collection for the fiscal year 2009/10 took place in November 2010 and February 2011. Within each district, we visited four government and two CHAM health centres. We randomly selected government facilities, while we purposively selected the mission health centres for convenience of data collection; they are located nearest to selected government health centres. At each facility, quantities of resources used were recorded. Data were collected on outputs of services, including number of visits, vaccinations, etc. This included visits for diagnosis of major disease categories (malaria, respiratory infection, diarrhoea, skin conditions, and malnutrition). We collected summary data if available; otherwise we extracted output data from facility records. Data on utilities and maintenance were collected either at the facility (for mission health centres) or at the district health office (for government health centres). Prices were collected from national suppliers or local retailers as appropriate. All prices reflect the fiscal year 2009/10, and were inflated, if needed, to and presented in 2010 US dollars, using an exchange rate of 151.75 [15]. We annualized the costs of capital items using a 3% discount rate [16].

We attempted to randomly sample 50 patient records each for outpatient and inpatient, if applicable, child under 5 visits at each facility. Without good data on the expected mean, variance, or difference in the cost of drugs between facilities, we did not perform sample size calculations. We extracted data related to age, sex, diagnosis, and the amount and type of drugs prescribed. Thirty-two per cent of sampled records were missing some data and we imputed missing data using multiple imputation by chained equations [17] and, in less than 1% of cases, standard treatment guidelines when this method failed. The fraction of missing information was less than 0.05 for 22 of the 24 facilities, and below 0.10 for the 2 remaining facilities; these patients were marked with dummy variables to assess whether they were outliers. Results reflect uncertainty due to imputation.

We used data from pharmacy records to estimate the quantities of drugs used only by children, namely the paediatric formulations of Lumefantrine/Arthemether (LA), all drugs in suspension, and vaccines. We used patient records to estimate the quantities for other drugs due to the uncertainty in allocating these drugs to U5s. Patient records were used to estimate less than 25% of total drug costs. Direct allocation [18] of other resources to children was based on staff interviews, time motion studies, and room usage within a facility. We did time motion studies for two hours in the morning and two hours in the afternoon targeting at least one provider per facility, with data collection staff observing providers from outside the consultation room. Time the provider spent during a child visit, time spent with other patients, and time spent doing other tasks were recorded.

Cost for overheads like vehicle use and training were based on the primary target of the training or use of vehicle; when this information is lacking, costs were allocated based on the proportion of visits from children to the total number of visits. Table 1 summarizes the data collected. Costs related to HIV/AIDS, dental care, and tuberculosis were excluded, either because they were separate programs from child health or they treated a negligible number of children. We interviewed up to 10 caretakers of children exiting each facility visited to ascertain their payments for accessing care and other experience with the visit, although these data are not directly used in the analysis.

**Table 1:** Data collected

**Parameter**	**District**				Average / Total*
	**1**	**2**	**3**	**4**	
Population	624,445	511,279	317,324	309,778	**440,707**
Estimated population Under 5	114,834	90,320	59,239	61,823	**81,554**
Number of Health Facilities	30	29	15	11	21
Number of health facilities sampled	6	6	6	6	24
% of health facilities sampled	20%	21%	40%	55%	**28%**
Population served of sample	186,666	162,763	128,507	129,326	607,262
% of district population served by sample	30%	32%	40%	42%	**36%**
Number of staff interviewed	12	12	12	11	47
Number of patient files sampled (of which for inpatient)	273 (0)	199 (0)	300 (0)	332 (32)	1104 (32)
Number of facilities with beds	6	6	5	5	22
Average number of beds among facilities with patient-beds (range)	4.0	5.2	7.2	6.4	**5.5**
	(2 to 10)	(2 to 11)	(2 to 12)	(2 to 12)	
Number of facilities with laboratory	0	0	2	1	3
Average number of clinical staff (range)	5.2	3.7	3.5	4.5	**4.2**
	(5 to 12)	(3 to 5)	(2 to 6)	(3 to 7)	

*Numbers in boldface are averages across the four districts, otherwise numbers reflect total values.

This study received approval from the Johns Hopkins University Bloomberg School of Public Health Ethical Review Committee and the National Health Research Committee in the Government of Malawi Ministry of Health. Oral informed consent was obtained from facility in-charges on the day of the visit, and all respondents gave oral informed consent before being interviewed and/or observed. The study was judged to have minimal risk to participants.

### Cost function specification

To analyse cost data, we regress the total costs of services against average prices, quantities of outputs, quality of services, type of facility, and case mix [19]. Each of these is discussed below. We check model specifications to determine if variables should be transformed using natural logs or if higher order terms should be included. Due to a low number of observations, interaction terms are not assessed. Sensitivity analysis is described and summarized in Additional file 1.

We collected staff salaries by grade at the district level, rather than by individual. However, different mixes of the types of staff at facilities, coupled with between district differences in salaries, mean that the average salary for clinical staff is different at all facilities observed, ranging from just under $2,200 to over $13,000. Thus, we include this in the model as an indicator of input prices.

Separate measures of at least three outputs could be included: the number of fully immunized children, the number of outpatient visits by children, and the number of inpatient admissions for children. Ideally, we would include emergency services, numbers fed through famine relief programs, and inpatient length of stay measures, but these data are not available. Since all facilities visited are first line health centres, we use outputs to measure the size and scope of services offered.

Quality is harder to observe, and is enhanced by verification of diagnosis and medication by an expert [20]. Lacking this data, we use data from patient records to estimate ‘the number of children correctly medicated’, which we define as the proportion of cases with a recorded diagnosis of a certain disease for whom the correct medication was also recorded as having been prescribed. The number of diseases available for analysis is limited. Three diseases, malaria, diarrhoea, and pneumonia/ARI, constitute over 60% of all cases and are used as indicator conditions for quality measurement. We assessed the data from patient records to ascertain if first or second line drugs were prescribed to children diagnosed with these diseases.

We use ownership to delineate facility type. Case mix is assessed as the percentage of visits due to particular diseases or severity of illnesses; we include the proportion of outpatient visits diagnosed with malaria because drugs for malaria are the most costly drug across facility types.

After running regressions of the form:

(1) $$\begin{array}{ll} \text{Total}\;\text{Cost} & =C+\beta_{1}{\text{Volume}}_{j}+\beta_{2}\text{Volume}-\text{Squared}\\ & +\beta_{3}\text{InputPrice}+\beta_{4}\text{Quality}\\ & +\beta_{5}\text{CaseMix}+\beta_{6}\text{Ownership}+\epsilon , \end{array}$$

we calculate the marginal cost as the expected change in cost when producing one more of a given type of output. Specifically marginal cost is calculated as:

(2) $$\text{dCost}/\text{dVolume}=\beta_{1}+\beta_{2}\text{Volume},$$

where Volume is set to the mean across facilities. In contrast, the average incremental cost is the change in costs from going from zero to the facility-mean, estimated by taking the difference between the predictions of the regression when Volume is set to zero and set to the mean for a given output, divided by the mean number of that output.

## Results

The bottom of Table 1 shows the characteristics of the facilities sampled. Only three of the facilities had laboratories; all three were CHAM facilities. Fifty-eight per cent of facilities had inpatient beds, with 5.5 beds on average in facilities with beds. Clinical staff employed at a facility ranged from two people to twelve people, with an average of 4.2 clinical staff present in a facility. Table 2 presents the total costs, outputs, and input variables, and Table 3 describes the breakdown of costs by facility type. The mean cost per patient, both inpatient and outpatient, seen for all health facilities was US$ 7.16 (95% CI: 5.24 to 9.08). CHAM facilities had an average cost of US$ 10.36 (95% CI: 4.92 to 15.80) per patient seen, while government facilities had an average cost of US$ 5.57 (95% CI: 4.52 to 6.60) per patient seen. Both total and average costs show a wide range, with total costs per facility ranging from about $16,000 to almost $200,000 and average cost per patient ranging just under $2.50 to over $10. When direct allocation is used to determine average costs for inpatient and outpatient care, the average cost of an outpatient visit was $5.36 and $7.89 at government and CHAM facilities, respectively, while an inpatient admission cost on average $15.94 and $39.22 for each type of facility. These costs were more variable at CHAM facilities than at government facilities. Staff remuneration and pharmaceuticals accounted for over 55% of total costs on average, and over 50% of costs at all but three facilities. LA generally accounted for over 75% of pharmaceutical costs.

**Table 2:** Summary of data used in analysis

**Variable**	**N**	**All facilities**		**Mean (standard error)**		**P >t**
		**Mean (standard error)**	**Range**	**CHAM facilities**	**Government health centres**	
	**(all facilities)**					
				**(n = 8)**	**(n=16)**	
Total costs (US$)	24	69,317	15,986 to	77,057	65,657	0.58
		(9,009)	192,174	(20,228)	(9,467)	
Outpatient visits for children U5	24	10,753	1,425 to	9,282	11,488	0.36
		(1,103)	22,156	(2,605)	(1,052)	
Inpatient admittances for children U5	24	133	0 to	117	140	0.86
		(60)	1,325	(51)	(88)	
Fully immunized children	24	915	0 to 2342	824	960	0.55
		(105)		(199)	(125)	
Average salary of clinical staff (US$)	24	5,205	2,194 to	6,540	4,536	0.06
		(502)	13,374	(1,202)	(390)	
Proportion of drugs out of stock (average of beginning and end of fiscal year)	24	0.28	0.07 to	0.17	0.33	0.0003**
		(0.02)	0.53	(0.02)	(0.02)	
Proportion of U5 outpatient visits diagnosed with malaria	23	0.54	0.05 to	0.48	0.57	0.38
		(0.05)	0.91	(0.10)	(0.05)	
Proportion of cases correctly medicated	22	0 .85	0.66 to	0.80	0.87	0.12
		(0.02)	0.97	(0.04)	(0.02)	

**Significant difference at p<0.01.

**Table 3:** Breakdown of costs by facility type

**Item**	**Facility type**	
	**Government health centres**	**CHAM**
**Inputs (Children U5)**		
*Variable*		
Staff remuneration	22%	39%
Training	9%	5%
Vehicle operation & Field visits	12%	11%
Non-medical supplies	1%	4%
Medical Supplies	6%	8%
Pharmaceuticals	38%	20%
Vaccines	2%	1%
Utilities & Maintenance	3%	2%
Lab & X-ray (variable)	--	1%
*Subtotal: Variable costs*	93%	91%
*Fixed*		
Building & Equipment (incl. lab/x-ray)	6%	5%
Vehicles	1%	4%
*Subtotal: Fixed costs*	7%	9%
***Average costs calculated with direct allocation (US$)***		
Outpatient visit (sd)	5.36	7.89
	(1.92)	(4.39)
Inpatient admission (sd)	15.94	39.22
	(7.60)	(17.57)

sd = standard deviation.

Donors directly financed 40% of costs at government health centres, and 21% of costs at CHAM facilities, not including funds provided through the SWAp. Pharmaceuticals and vaccines accounted for 78% of donor support at government health centres and 70% at CHAM facilities. Donor procurement of LA was the most important component of this support.

Government and CHAM facilities had similar utilization numbers, with an average of 10,753 child outpatient visits (range among facilities: 1,425 to 22,156), over 100 inpatient admittances (range: 0 to 1,325), and about 900 fully immunized children (range: 0 to 2,342) with no statistically significant differences between the two facility types.

On average, 27% of drugs used for children present at facilities at some point during the fiscal year were out of stock at the two time points measured (beginning or end of the fiscal year). This was 17% at CHAM facilities and 33% at government health centres (p<0.01). An average 54% of outpatient U5 cases were diagnosed with malaria among the 26 facilities with this data. However, malaria was diagnosed by observation and the proportion of cases diagnosed with malaria ranges from 5% to 91% between facilities, calling into question the accuracy of the diagnoses.

Regression analyses were done on total costs in natural units, because the data in the raw scale had relatively low skew and a kurtosis coefficient under 3 [21] (See Additional file 2). Four regression specifications are presented in Table 4. Proportion of drugs out of stock, the proportion of cases diagnosed with malaria, average salary of clinical staff, and the proportion of cases correctly medicated did not improve model fit in any of the models (p<0.10) (data not shown). Outputs and the indicator for CHAM facilities are only statistically significant in the specification that includes the proportion of drugs out of stock. The direction of the coefficient for the proportion of drugs out of stock is positive. This finding is difficult to interpret; perhaps having cheaper first line drugs in stock lowers costs, or, because CHAM facilities are more likely to have drugs in stock, this variable could be capturing higher CHAM costs. The number of fully immunized children does not improve model fit. All other variables, including those assessing missingness, do not improve model fit (p<0.10). The preferred specification is Model 4, which had the highest R-squared and log likelihood. We use this model for the remainder of the analyses, although we report average and marginal cost estimates for all models to facilitate comparisons. Results appear to be robust based on sensitivity analyses (see Additional file 1).

**Table 4:** Results of regression

**Variable**	**Beta coefficients (standard error)**			
				**Preferred**
	**Model 1**	**Model 2**	**Model 3**	**Model 4**
Outpatient visits	16.5 (10.2)	13.0 (10.5)	16.6 (10.5)	22.6 (8.5)*
Square of outpatient visits	-0.001	-0.001	-0.01 (0.001)	-0.002
	(0.001)	(0.001)		(0.001)*
Cube of outpatient visits	4.0 × 10^-8^	4.0 × 10^-8^	4.0 × 10^-8^	7.0 × 10^-8^
	(3.0 × 10^-8^)	(3.0 × 10^-8^)	(3.0 × 10^-8^)	(2.0 × 10^-8^)*
Inpatient admissions	23.7 (21.4)	26.9 (21.3)	17.2 (66.2)	13.0 (17.6)
Square of inpatient admissions			0.006 (0.05)	
Fully immunized children		16.2 (13.1)		
CHAM facility	18,548	19,294	18,978	46,425
	(14,283)	(14,088)	(15,263)	(14,374)**
Proportion of drugs out of stock				1,862 (573)**
Constant	-19,425	-20,249	-19,328	-87,976
	(35,242)	(34,737)	(36,264)	(35,425)*
N (Facilities)	24	24	24	24
Adjusted r-squared	0.625	0.636	0.603	0.755
Log likelihood	-275.5	-274.4	-275.4	-269.7
Likelihood ratio test: p > chi2 compared to model 1	N/A	0.150	0.902	0.0007**

Dependent variable = Total facility cost of child health services for fiscal year 2009/2010 in US dollars (natural units).

*significant at p<0.05.

**significant at p<0.01.

Table 5 presents the estimated marginal cost and average incremental cost of services. One more inpatient admission, for example in model 4, is associated with an increase of $13.0 in total costs, which is also the average incremental cost. For outpatient visits, the marginal cost is estimated at under $1 in the fourth model. The models show that marginal costs for outpatient visits are lower than average incremental costs.

**Table 5:** Elasticity, per visit costs, and economies of scale at government health centres and CHAM facilities at mean values

**Model**	**Outpatient visits**			**Inpatient admissions**		
	**E**	**MC**	**AIC**	**E**	**MC**	**AIC**
*Government health centres*						
1	0.53	2.81	6.68	0.05	23.7	
2	0.49	2.60	5.41	0.06	26.9	
3	0.53	2.79	6.68	0.04	18.8	18.0
4	0.16	0.82	7.20	0.03	13.0	
*CHAM Facilities*						
1	0.34	2.66	7.64	0.04	23.7	
2	0.30	2.37	6.12	0.04	26.9	
3	0.34	2.64	7.65	0.03	18.5	17.9
4	0.10	0.79	8.76	0.02	13.0	

E = Elasticity.

MC = Marginal incremental cost.

AIC = average incremental cost.

**Figure 1:**
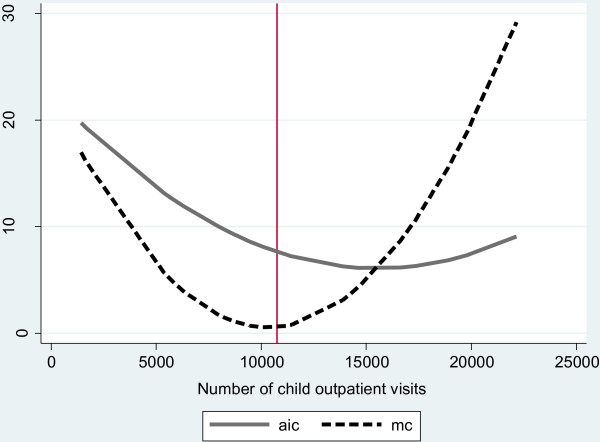
Estimated average incremental cost and marginal cost for outpatient visits*. aic = average incremental cost mc = marginal incremental cost vertical line = average number of outpatient visits in the observed sample. *This graph is constructed holding all variables at their mean across facilities except for the number of child outpatient visits. We then predict the expected aic and mc for the outpatient volume observed at all facilities in our sample. The presented lines connect these predicted values.

Figure 1 presents the estimated average incremental cost and marginal cost curves, based on the fourth model, for outpatient visits across the range of outpatient visits observed. The mean number of outpatient visits observed in the sample is close to the lowest marginal incremental cost, suggesting that average incremental costs could be lowered if health facilities served more children at the current level of quality.

In order to understand the cost profile at different scales, in Table 6 we summarize the proportion of total costs by cost category for three levels of outpatient utilization: low utilization under 7,000 outpatient visits, mid-level utilization of 7,000 to 14,999 visits, and high utilization of 15,000 visits and over.

**Table 6:** Breakdown of costs by facility utilization

**Item**	**Facility utilization level**		
	**Low utilization (Fewer than 7,000 outpatient visits)**	**Middle utilization (7,000 to 14,999 outpatient visits)**	**High utilization (Over 15,000 outpatient visits)**
Number of health centres	6	13	5
of which CHAM	4	2	2
Population served (average)	18,345	24,372	36,071
Estimated Children U5 (average)	3,385	4,597	6,561
**Outputs (Children U5)**			
Inpatient admissions (average)	71	82	338
Outpatient visits (average)	4,446	10,507	18,961
FIC (average)	495	1,013	1,165
Child outpatient utilization rate	1.31	2.29	2.89
Proportion of caretakers reporting over 1 hour travel time to facility (95% CI)*	67%	60%	56%
	(52 – 81%)	(47 – 73%)	(41 – 71%)
**Inputs (Children U5)**			
*Variable*			
Staff remuneration	47%	23%	29%
Average number of clinical staff	3.7	3.8	5.8
Outpatient visits per clinical staff	1213	2732	3269
Average number of staff (all)	37.2	29.8	45.6
Outpatient visits per staff (all)	120	352	416
Training	5%	8%	6%
Vehicle operation & Field visits	6%	7%	20%
Non-medical supplies	1%	2%	3%
Medical Supplies	8%	7%	5%
Pharmaceuticals	18%	42%	22%
Average pharmaceutical spending per visit ($)	1.4	2.6	1.3
Proportion of drugs out of stock	0.19	0.32	0.28
Proportion of children correctly medicated	0.59	0.82	0.81
*Subtotal: Variable costs*	87%	94%	92%
*Fixed*			
Building & Equipment (incl. lab/x-ray)	8%	5%	5%
Vehicles	5%	1%	3%
*Subtotal: Fixed costs*	13%	6%	8%

*Based on 229 exit interviews of caretakers at facilities; 60 respondents for low utilization facilities, 128 respondents for middle utilization facilities, and 41 respondents for high utilization facilities. Difference between low utilization and high utilization facilities is statistically significant (p<0.05) in logit regression when controlling for district (data not shown).

### Discussion

We find that an average health centre in the four selected districts in Malawi is not operating at an optimal scale of services. Table 6 shows that high utilization facilities spent 20% of their total budget, on average, for vehicle operation and field visits. On the other hand, staff and clinical staff at these facilities see more children than at other facilities, and have the highest number of visits per child in their catchment area per year. Because high-utilization facilities are more likely to have vehicles than other facilities, the upward slope of marginal costs at high utilization levels indicated by the regression probably reflects the cost of vehicle operation rather than diseconomies of scale with respect to staff or other fixed costs. Thus, the increasing average costs for facilities over 15,000 visits should be interpreted with caution, and our models should include another output for outputs related to vehicle use.

Facilities with low levels of utilization also tend to have both a smaller population of children in their catchment area and a lower number of visits per child per year. Thus, these facilities have lower number of visits per staff and per clinical staff, but these facilities also have a greater number of non-clinical staff than facilities with mid-levels of utilization. We also find, based on exit interviews, that a greater proportion of caretakers reported traveling over 1 hour to receive care among low-utilization facilities compared with high utilization facilities. This may indicate that low-utilization facilities are also located in areas with a lower population density. These findings may be confounded by the fact that 4 of the 6 low utilization facilities are CHAM health centres, and user fees may lower demand. However, for the two government-operated low utilization facilities, the average utilization rate is only 1.89 visits per child per year. Thus, we cannot draw conclusions as to the reasons for low utilization, but note that it appears to be associated with lower levels of efficiency.

Likely, active steps are needed to increase demand and utilization at these health centres; facilities with low utilization (e.g., under 7,000 children in the catchment area or with utilization rates under 2 visits per child per year) would especially benefit from these activities. Increasing utilization rates would not fully help low utilization facilities alleviate their inefficiency with respect to scale since they serve a smaller number of children; these facilities also appear most likely to be affected by community-based case management since they are possibly in more remote areas. The government, which has goal of having the 80% of the population live within 5 kilometres of a health facility [20], may be willing to bear this loss of efficiency, but expansion of non-child services is another possible way to alleviate inefficiency. Staffing patterns may bear scrutiny; however, there may be reasons health facilities with smaller catchment populations to have more non-clinical staff; e.g., non-clinical staff may be used for outreach.

While CHAM facilities charge clients user fees, we find, on average, their volume of visits is similar to government health centres. Government health centres received, on average, 2.5 visits per child in their catchment area in the year observed, while CHAM facilities received 2.2 visits per child per year. We found, however, that 72% of all outpatient visits at CHAM facilities were from children, compared with 36% at government health centres. This helps explain why, despite similar levels of outputs, CHAM facilities are more expensive; their fixed and semi-fixed costs are, overall, spread across fewer visits.

Our data do not measure quality well, but we found that around 85% of children were prescribed a medicine that was consistent with their recorded diagnosis. In an observation-based study conducted in 2009, researchers found that 53.6% of children with pneumonia, 63.4% of children with malaria, and 43% of children with dehydration were given the correct prescription [22]. These rates, which were accompanied by confirmation of diagnosis, are substantially lower than in our data. Thus, there may substantial room to improve the quality of care, which is a second path to increasing the efficiency of services. We also found a large proportion of drugs were out of stock at government health centres, and that higher stock-out rates were associated with higher costs. This is another area where quality improvement may improve cost profiles.

This study suffers from a number of weaknesses. Although 1104 patient records were analysed the sample size of facilities is only 24, and deriving conclusions from small sample sizes is problematic. For example, based on parameter uncertainty in the fourth model, the optimal scale of services could as low as 5000 outpatient visits per facility or above the highest number of outpatients visits observed at any health centre (data not shown), and deriving benchmark figures for optimal utilization requires more study. Further, our survey only covered four of Malawi’s 28 districts, making it difficult to generalize about all of Malawi. (In Additional file 3 we provide a comparison of basic indicators for the 4 districts selected and for Malawi as a whole showing that the 4 districts were actually quite representative in their health statistics). Further, the quality of the data was not always optimal. Often, confirmation of data, e.g., in registries, found discrepancies with officially reported data. In some cases, records were missing or incomplete. However, the results appear robust to moderate levels of data error (see Additional file 3).

Assessing how costs change over time, and how they change in relation to new programmes, is important both to provide feedback to facility and district managers and to inform projections of the amount of resources needed to achieve health and mortality reduction goals. We plan to conduct a follow-up study at the same facilities after the community-based case management program has matured.

## Competing interests

The authors declare that they have no financial or personal conflicts of interest.

## Authors’ contributions

BJ contributed to the study design, data collection, analysis, and drafted the manuscript. SM contributed to the study design, data collection, analysis, and commented on the manuscript. DW and WM contributed to the study design and commented on the manuscript. DB contributed to the study design, analysis, and commented on the manuscript. All authors approved the final manuscript.

## Supplementary Material

Additional file 1 Description and summary of sensitivity analysis. Click here for file

Additional file 2 Summary data for outcome variables. Click here for file

Additional file 3 **Comparison of basic indicators for sampled districts and for Malawi as a whole **[23]**-**[25]**.** Click here for file
