# Supplementary material for: A cost function analysis of child health services in four districts in Malawi
Source: Cost Eff Resour Alloc. 2013 May 10;11:10. doi: 10.1186/1478-7547-11-10 (PMC3729666; doi:10.1186/1478-7547-11-10)
Supplement: Additional file 1 — Description and summary of sensitivity analysis. [file 1478-7547-11-10-S1.doc]

*Annex 1: Description and summary of sensitivity analysis*

We conducted sensitivity analyses in three ways. First, models are assessed using robust standard errors to account for possible clustering at the district level to determine if coefficients lose statistical significance. Second, because of uncertainty in the costs of drugs due to sampling and imputation of missing data, high and low estimates of the costs of pharmaceuticals were calculated based on 95% confidence intervals and different methods of calculating total costs (i.e., sample of patient records and pharmacy records). Regressions are re-run using these high and low estimates of costs to determine if the magnitude and statistical significance of the estimated coefficients in the model change. Finally, due to concerns over the quality of the data available for the variables on the right hand side of the equation, we drew 100 random values from a uniform distribution of ±10% of the observed value for each variable, 100 random values from a uniform distribution of the plausible high and low values for the total cost of each facility, and ran the regressions on these data to determine plausible ranges for the estimated coefficients and the proportion of regressions where the coefficient is statistically significant at p<0.05. Uniform distributions are used to represent a plausible range of measurement error at each facility.

**Annex 1, Table 1: Range of results of regression models based on high and low total costs**

| **Variable** | **Beta coefficients** | |
| --- | --- | --- |
|  | Model 4: High and low total cost estimates only | Model 4: Simulation with range of total costs and independent variables (percentage of models where p(t)<0.05) |
| Outpatient visits | 16.3 to 23.1*** | 20.8 to 22.5  (83%) |
| Square of outpatient visits | -0.002 to -0.002*** | -0.002 to -0.002 (57%) |
| Cube of outpatient visits | 7.0 x 10^-8^  to 7.0 x 10^-8^*** | 6.0 x 10^-8^  to 7.0 x 10^-8^  (85%) |
| Inpatient admissions | 9.0 to 20.0* | 12.3 to 17.5  (1%) |
| CHAM facility | 23,263 to 47,976** | 45,762 to 47,390 (100%) |
| Proportion of drugs out of stock | 85,644 to 186,230** | 173,617 to 180,296 (99%) |

*** Significant at p<0.05 in all three models

** Significant at p<0.05 in two of three model

* Significant at p<0.05 in only one model

Sensitivity analyses are shown in Annex 3 Table 1 and generally confirm the robustness of model fit with respect to outpatient visits. The coefficients for inpatient admissions and CHAM have a greater range of values, although conclusions about their statistical significance do not change greatly. When robust standard errors are used to account for clustering at the district level, the model coefficients for outpatient visits lose significance in the fourth model, while the coefficient for inpatient admissions gains significance in all models except the second model.

## In the simulation exercises, the range of coefficients found do not vary greatly (see the second column of Annex 3 Table 1) except possibly in the case of inpatient admissions. Further, variables not found to be statistically significant are not likely to be significant in the simulations, while variables found to be statistically significant in the models were likely to remain significant (results for all models not shown). This indicates that the results are not likely to be affected by measurement error.
