# Supplementary material for: A cost function analysis of child health services in four districts in Malawi
Source: Cost Eff Resour Alloc. 2013 May 10;11:10. doi: 10.1186/1478-7547-11-10 (PMC3729666; doi:10.1186/1478-7547-11-10)
Supplement: Additional file 2 — Summary data for outcome variables. [file 1478-7547-11-10-S2.doc]

## Annex 2: Summary data for outcome variables

| Variable | Mean | Standard deviation | Min | Max | Skew | Kurtosis | For residuals after regression | |
| --- | --- | --- | --- | --- | --- | --- | --- | --- |
|  |  |  |  |  |  |  | Skew | Kurtosis |
| Total cost of child health services for the year (2010 US$) | 69,317 | 44,136 | 15,986 | 192,174 | 1.27 | 3.90 | -0.36 | 2.94 |
| Ln(Total cost of child health services for the year) | 10.97 | 0 .60 | 9.68 | 12.17 | 0.07 | 2.68 | -0.03 | 1.48 |
