# Supplementary material for: A cost function analysis of child health services in four districts in Malawi
Source: Cost Eff Resour Alloc. 2013 May 10;11:10. doi: 10.1186/1478-7547-11-10 (PMC3729666; doi:10.1186/1478-7547-11-10)
Supplement: Additional file 3 — Comparison of basic indicators for sampled districts and for Malawi as a whole [23]-[25]. [file 1478-7547-11-10-S3.doc]

## Annex 3: Comparison of basic indicators for sampled districts and for Malawi as a whole

| **Parameter** | **Average for 4 districts sampled** | **Average for Malawi** |
| --- | --- | --- |
|
| Population [23] | 440,707 | 408,661 |
| % of population urban [23] | 3.8% | 15.3% |
| % of population under 5 years of age [23] | 18% | 18% |
|  |  |  |
| Percentage of children fully immunized [24] | 86.8% | 80.9% |
| % of children interviewed with fever [24] | 59% | 59% |
| % of diarrhoea cases seeking care [24] | 59.9% | 57.3% |
| % of diarrhoea cases treated with ORS (among those seeking care) [24] | 64.3% | 69% |
| % of children with any anaemia [24] | 64.3% | 63.5% |
|  |  |  |
| Under 5 mortality [25] | 158 | 140 |
